# Artesunate nanoliposome-hydrogel: a dual-modal therapy for post-surgical melanoma

**DOI:** 10.7150/thno.120793

**Published:** 2026-01-01

**Authors:** Hongmei Chen, Zhongke Wang, Ying Huang, Aodi Li, Xinlei Xiong, Yifan Pu, Ling Guo

**Affiliations:** 1 School of Stomatology, Southwest Medical University, Luzhou, China.; 2 Luzhou Key Laboratory of Oral & Maxillofacial Reconstruction and Regeneration, Luzhou, China.; 3 Department of Prosthodontics, The Affiliated Stomatological Hospital of Southwest Medical University, Luzhou, China.

**Keywords:** Melanoma, Post-surgical recurrence, Wound regeneration, Artesunate, Mitochondrial apoptosis

## Abstract

**Background:** Melanoma management faces the dual challenge of preventing tumor recurrence while ensuring optimal post-surgical wound healing, particularly problematic given melanoma's high recurrence rates and therapeutic resistance. Artesunate (ARS) emerges as a promising multimodal agent with concurrent anticancer, anti-inflammatory, and tissue-regenerative properties. However, its anti-melanoma mechanisms remain incompletely characterized, and clinical translation has been limited by suboptimal pharmacokinetics.

**Methods:** We employed transcriptomic profiling (RNA-seq) to identify novel ARS-regulated pathways. Subsequently, we developed an optimized drug delivery system comprising: amphiphilic nanoliposomes for efficient ARS encapsulation and enhanced cellular internalization, and a carboxymethyl chitosan hydrogel matrix (ARS-LS-Gel) engineered to provide sustained drug release while promoting tissue regeneration. Comprehensive physicochemical characterization preceded systematic *in vitro* evaluation in melanoma (B16F10, A375) and normal cell models, assessing cytotoxicity, cellular uptake, and mechanistic pathways. Dual efficacy was quantified *in vivo* using syngeneic melanoma and full-thickness wound healing models.

**Results:** The ARS-LS-Gel system demonstrated optimal physicochemical characteristics, including well-dispersed particles, sustained drug release kinetics and exceptional biocompatibility. It potently induced melanoma cell apoptosis through p53-mediated mitochondrial dysfunction, characterized by: (1) sustained ROS accumulation, (2) cytochrome C release, (3) mitochondrial membrane potential collapse, (4) impaired ATP synthesis, and (5) calcium overload. *In vivo*, the platform significantly suppressed tumor progression, evidenced by enhanced apoptosis and reduced Ki-67 expression. Concurrently, it accelerated wound regeneration via targeted downregulation of pro-inflammatory mediators (TNF-α, IL-1β) and enhanced collagen deposition.

**Conclusion:** The ARS-LS-Gel platform's ability to simultaneously address oncogenic progression and tissue repair represents a significant conceptual and practical advancement in post-surgical cancer management. By bridging fundamental mechanistic discovery with engineered therapeutic delivery, our findings provide a robust foundation for imminent translational development in melanoma therapy and beyond.

## Introduction

Melanoma is a highly aggressive tumor arising from melanocytes in cutaneous and extracutaneous tissues, and has shown increasing incidence among younger populations in recent years 1. The diagnostic challenge posed by its morphological similarity to common moles and benign lesions leads to delayed diagnosis, with most patients presenting at advanced stages 2. While surgical excision remains the primary treatment, it often results in difficult-to-repair defects and inadequate tumor clearance due to standard margins 3, with studies reporting postoperative recurrence or metastasis in up to 80% of patients 4. Conventional approaches like postoperative adjuvant therapy aim to eliminate residual tumor cells and prevent recurrence 5. However, current first-line chemotherapies face increasing drug resistance 6, contributing to a <5-year survival rate in fewer than 10% of advanced cases 7. Moreover, chemotherapy-induced adverse effects impede postoperative wound recovery 8. Thus, novel therapeutic strategies are urgently required to improve melanoma outcomes.

Artesunate (ARS), a water-soluble semi-synthetic derivative of artemisinin, serves as a first-line antimalarial agent in clinical practice 9. Emerging evidence highlights artemisinin-based drugs for their unique antitumor mechanisms, such as oxidative stress induction 10, 11, apoptosis/autophagy activation, and ferroptosis initiation 12, 13. Artemisinin derivatives demonstrate broad-spectrum antitumor activity, suppressing proliferation in breast cancer, leukemia, and hepatocellular carcinoma models 14-17. Notably, ARS exerts anti-melanoma effects via proliferation inhibition, angiogenesis suppression 10, 18, and metastasis blockade 19, offering therapeutic promise for drug-resistant cases. ARS also exhibits potent anti-inflammatory properties, mediated through inflammatory cytokine suppression, immune cell modulation, and NF-κB/TLR-4/ERK/MAPK pathway interference 20, 21. Furthermore, ARS synergizes with physiotherapy to accelerate wound healing via scar formation reduction 22. However, its clinical potential is limited by poor water solubility 23, requiring advanced delivery systems for optimal therapeutic application.

In recent years, nano-drug delivery systems have made remarkable progress in the field of drug delivery, offering diverse solutions to improve drug solubility, enhance targeting capability, and reduce toxic side effects. Among various drug carriers, polymeric nanoparticles 24, nanovesicles 25, and LC3-based autophagosome tumor vaccine delivery systems 26, each possess distinct characteristics while facing certain limitations, such as burst release effects and manufacturing challenges. In contrast, nanoliposome-hydrogel drug delivery systems demonstrate unique advantages in loading hydrophobic drugs.

Liposome-based delivery systems, composed of phospholipid bilayer vesicles, offer an effective solution for ARS encapsulation and tumor delivery 27. Their biomimetic structure enables efficient cellular uptake through membrane fusion and endocytic pathways 28, significantly improving drug accumulation and retention in tumors 29. However, conventional liposomes show limitations, including burst release and heterogeneous drug distribution 30. Incorporation into hydrogel matrices, particularly those based on carboxymethyl chitosan (CMCS), can overcome these challenges by providing sustained release and structural support 31. The CMCS hydrogel's unique properties - including pH-dependent charge characteristics, enhanced permeability, and extracellular matrix-mimicking structure - make it particularly suitable for combined drug delivery and tissue regeneration 32, 33.

**Scheme 1:**
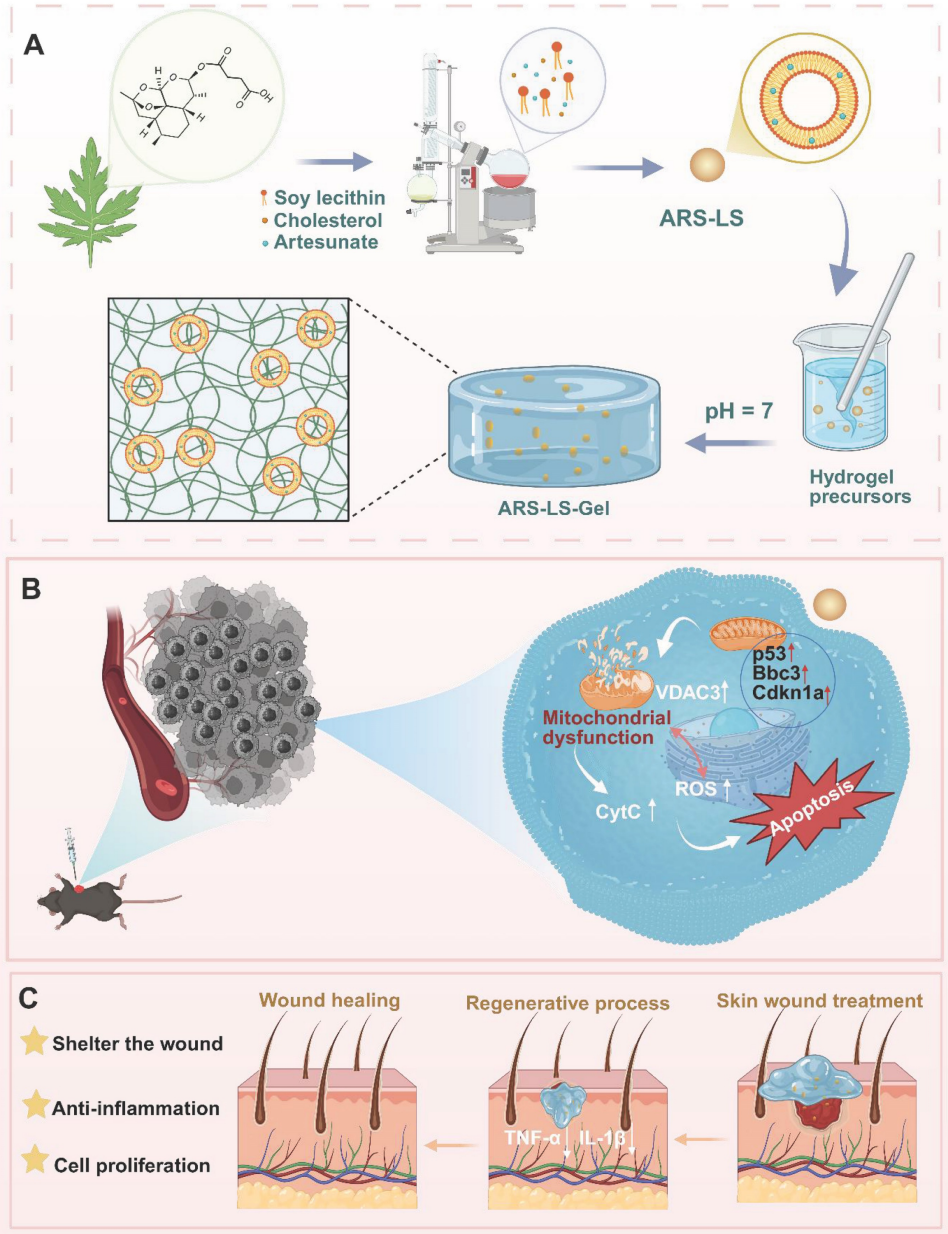
The schematic depicts the preparation and application of ARS-LS-Gel for postoperative melanoma recurrence management and wound healing enhancement. Preparation of ARS-LS-Gel (A), and it inhibits melanoma via p53-mediated apoptosis (B), enhances wound healing by reducing inflammation and boosting collagen (C).

Our study first employed whole transcriptome sequencing to reveal that ARS exerts potent anti-melanoma effects primarily through p53-mediated mitochondrial dysfunction. Based on this mechanistic insight, we developed an innovative ARS delivery platform (ARS-LS-Gel) (Scheme 1), combining amphiphilic nanoliposomes for efficient encapsulation and tumor cell delivery, and a CMCS hydrogel matrix, providing two key functions: sustained drug release and wound protection through its porous structure. This integrated system not only enhances ARS's tumoricidal effects but also synergistically downregulates pro-inflammatory cytokines (TNF-α, IL-1β) to promote wound healing. The resulting dual-functional platform represents a significant advance in post-surgical melanoma management, addressing both tumor recurrence and wound repair through a single therapeutic strategy.

## Results and Discussion

### RNA-seq and data analysis

**Figure 1:**
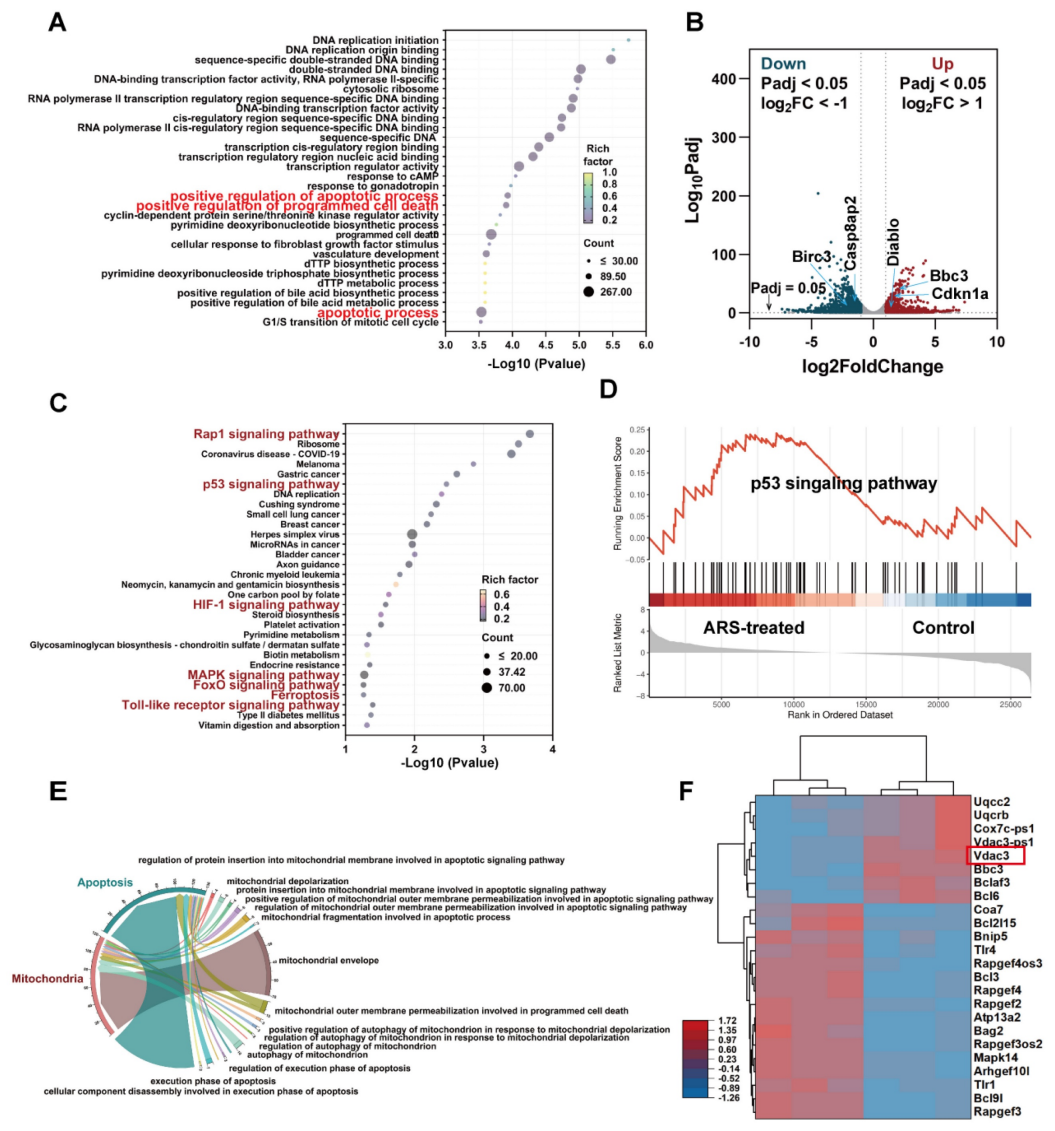
Transcriptomic analysis of apoptosis mechanisms in ARS- versus PBS-treated B16F10 cells (n = 3). (A) GO enrichment analysis. (B) Volcano plot illustrating the DEGs in the control and treated groups. (C) KEGG pathway enrichment analysis highlighting apoptosis-related signaling pathways. (D) GSEA analysis targeting the p53 signaling pathway. (E) Chord diagram demonstrating functional relationships between mitochondrial processes and apoptotic pathways. (F) Heatmap visualization of mitochondrial function-related gene expression patterns.

To elucidate the anti-tumor mechanisms of ARS, we conducted differential gene expression analysis comparing ARS-treated and untreated B16F10 cells. Bulk RNA-seq revealed 3,710 differentially expressed genes (DEGs) (1,573 upregulated, 2,137 downregulated; |log2FC| ≥ 1, FDR < 0.05) (Figure S1). Gene Ontology (GO) enrichment analysis demonstrated significant association of these DEGs with key apoptotic processes, including "positive regulation of apoptosis," "programmed cell death," and "apoptotic process" (Figure 1A). Volcano plot analysis identified significant upregulation of pro-apoptotic genes (Bbc3, Cdkn1a, Diablo) and downregulation of anti-apoptotic genes (Casp8ap2, Birc3) (Fig. 1B). KEGG pathway analysis of the top 30 enriched pathways implicated several apoptosis-related signaling cascades, including: p53 signaling pathway/Rap1 signaling pathway/HIF-1 signaling pathway/MAPK signaling pathway/FoxO signaling pathway/Ferroptosis/Toll-like receptor signaling pathway (Figure 1C).

The p53 pathway emerged as a central regulator of ARS-induced apoptosis 34-36, supported by GSEA (Figure 1D). Notably, we found that the above upregulated genes Bbc3 and Cdkn1a (p21) were closely related to the p53 signaling pathway. Bbc3 (a direct p53 transcriptional target) induces mitochondrial apoptosis 37, 38, while CDKN1A promotes apoptosis through mitochondrial membrane permeability modulation 39. The p53-mediated apoptotic pathway exhibits intimate mechanistic connections with mitochondrial function 40, 41. As the cellular powerhouses responsible for energy production, calcium homeostasis, and iron metabolism, mitochondria serve as central regulators of intrinsic apoptosis through cytochrome C (Cyt C) release 42, 43.

Further transcriptomic analysis confirmed mitochondrial involvement (Figure 1E), with altered expression of regulators such as VDAC3 (Figure 1F), a subtype of the voltage-dependent anion channel (VDAC), which primarily forms aqueous pores on the outer mitochondrial membrane (OMM). These channels facilitate the exchange of essential metabolites and molecules, playing a pivotal role in cellular metabolism and homeostasis 44, and are closely associated with the pathogenesis and progression of mitochondrial diseases 45.

As a key component of the mitochondrial permeability transition pore (mPTP), VDAC3 dysfunction can lead to mitochondrial membrane potential (MMP) impairment and mitochondrial swelling 46, ultimately triggering Cyt c release. Studies have demonstrated that VDAC3 overexpression amplifies mPTP opening, exacerbates Cyt c release, and consequently induces apoptosis 47, 48. These findings suggest that ARS may trigger apoptosis via p53-mediated mitochondrial dysfunction.

### Synthesis and characterization of ARS-LS-Gel

**Figure 2:**
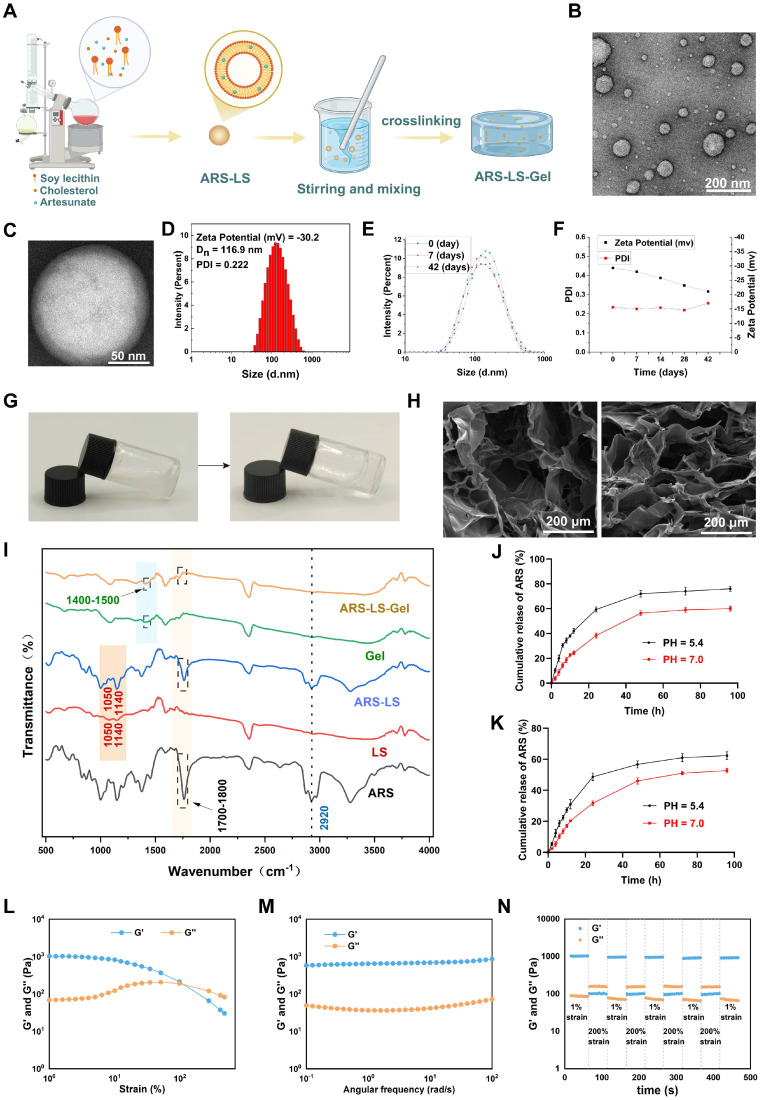
Characterization of ARS-LS-Gel nanocomposite. (A) Schematic illustration of the ARS-LS-Gel preparation process. (B) TEM image showing the spherical morphology of ARS-LS nanoparticles (scale bar: 200 nm). (C) High-magnification TEM image revealing the characteristic bilayer structure of ARS-LS (scale bar: 50 nm). (D) Physicochemical characterization of ARS-LS, including zeta potential, hydrodynamic diameter, and polydispersity index (PDI). (E) Particle size stability profile at 0, 7, and 42 days. (F) Stability monitoring of zeta potential and PDI over 42 days. (G) Macroscopic appearance of the hydrogel precursor solution (left) and formed gel (right). (H) SEM images comparing the porous structures of blank hydrogel (left) and ARS-LS-Gel (right) (scale bar: 200 μm). (I) FTIR spectra analysis of ARS-LS-Gel and its individual components. (J) ARS release from ARS-LS under different pH conditions, data presented as mean ± SD (n = 3). (K) ARS release from ARS-LS-Gel under different pH conditions, data presented as mean ± SD (n = 3). (L) Strain sweeps of ARS-LS-Gel. (M) Frequency sweeps of ARS-LS-Gel. (N) Step-strain tests of ARS-LS-Gel.

Figure 2A illustrates the fabrication of ARS-LS-Gel. ARS-LS appeared as a translucent suspension (Figure S2), with TEM showing spherical nanoparticles (Figure 2B) and a phospholipid bilayer structure (Figure 2C). Particle size averaged 116.5 ± 0.45 nm, with a zeta potential of -29.3 ± 1.1 mV and PDI of 0.234 ± 0.011 (Figure 2D). Under 4 °C storage, ARS-LS exhibited excellent stability over 42 days, maintaining consistent size, zeta potential (< -20 mV), and PDI (< 0.3) (Figure 2E-F).

Following pH adjustment to neutrality with triethanolamine, the solution of ARS-LS-Gel underwent a sol-gel transition accompanied by a visible change from turbid to transparent (Figure 2G). As shown in the attached results (Figure S3), the hydrogel allows clear visualization of underlying letters (A, B, C) with comparable clarity to unobstructed controls (Letter D), confirming its high transparency. Scanning electron microscopy (SEM) characterization of both blank hydrogel and ARS-LS-Gel revealed three-dimensional porous networks with uniform pore distribution (Figure 2H), which facilitates the diffusion of drugs, oxygen, and intracellular metabolites, providing favorable conditions for cell growth and migration 49. Additionally, a measurable reduction in average pore diameter following ARS-LS incorporation, likely attributable to intermolecular interactions (van der Waals forces and hydrogen bonding) between the liposomal components and hydrogel matrix 50.

Fourier-transform infrared spectroscopy (FTIR) analysis confirmed the structural integrity of ARS-LS-Gel, revealing characteristic vibrational modes including phospholipid signatures at 1050 cm⁻¹ (P=O stretching) and 1140 cm⁻¹ (PO₃^2-^ symmetric stretching) 51, 52, cholesterol C-H stretching at 2920 cm⁻¹, and carboxylate formations (1400 - 1450 cm⁻¹) from neutralization reactions between carboxymethyl chitosan/carbomer 940 and triethanolamine. Critically, the preservation of artesunate's diagnostic peaks 53. including carbonyl (C=O) stretching between 1700-1800 cm⁻¹ and aliphatic C-H stretches at 2920 cm⁻¹ confirmed intact drug incorporation without chemical degradation (Figure 2I).

Our systematic evaluations using the validated absorbance method revealed that the ARS-LS formulation achieved an encapsulation efficiency of 74.03% ± 1.98% with a drug loading rate of 22.28% ± 3.14%, while the ARS-LS-Gel composite demonstrated slightly reduced but still excellent values at 71.61% ± 2.46% encapsulation efficiency and 14.76% ± 2.56% drug loading. The minor decrease in values for the gel formulation reflects the expected additional mass contribution from the hydrogel matrix while maintaining strong drug retention capability. Subsequently, we systematically evaluated the pH-dependent release kinetics of ARS from both nanoplatforms under simulated physiological (PBS pH 7.0) and tumor microenvironment (PBS pH 5.4) conditions. As shown in (Figure 2J-K), ARS-LS-Gel demonstrated superior sustained release characteristics compared to ARS-LS, particularly at tumor pH (5.4), where electrostatic interactions between protonated carboxymethyl chitosan and liposomes further slowed drug diffusion.

Rheological analysis confirmed the hydrogel's robust mechanical properties. Strain sweeps showed solid-like behavior (G′ > G″) with a critical strain of 100%, indicating high deformation resistance (Figure 2L). Frequency sweeps revealed stable G′ under physiological shear (0.1-100 rad/s), ensuring dynamic adaptability (Figure 2M). Step-strain tests demonstrated rapid self-healing: after 200% strain (beyond the critical limit), G′ dropped from 1000 to about 100 Pa but fully recovered within seconds upon returning to 1% strain (Figure 2N). These results highlight ARS-LS-Gel's optimal balance of stiffness, stress tolerance, and instant self-recovery—key for wound-healing applications.

### *In vitro* cell compatibility test and anti-tumor function

**Figure 3:**
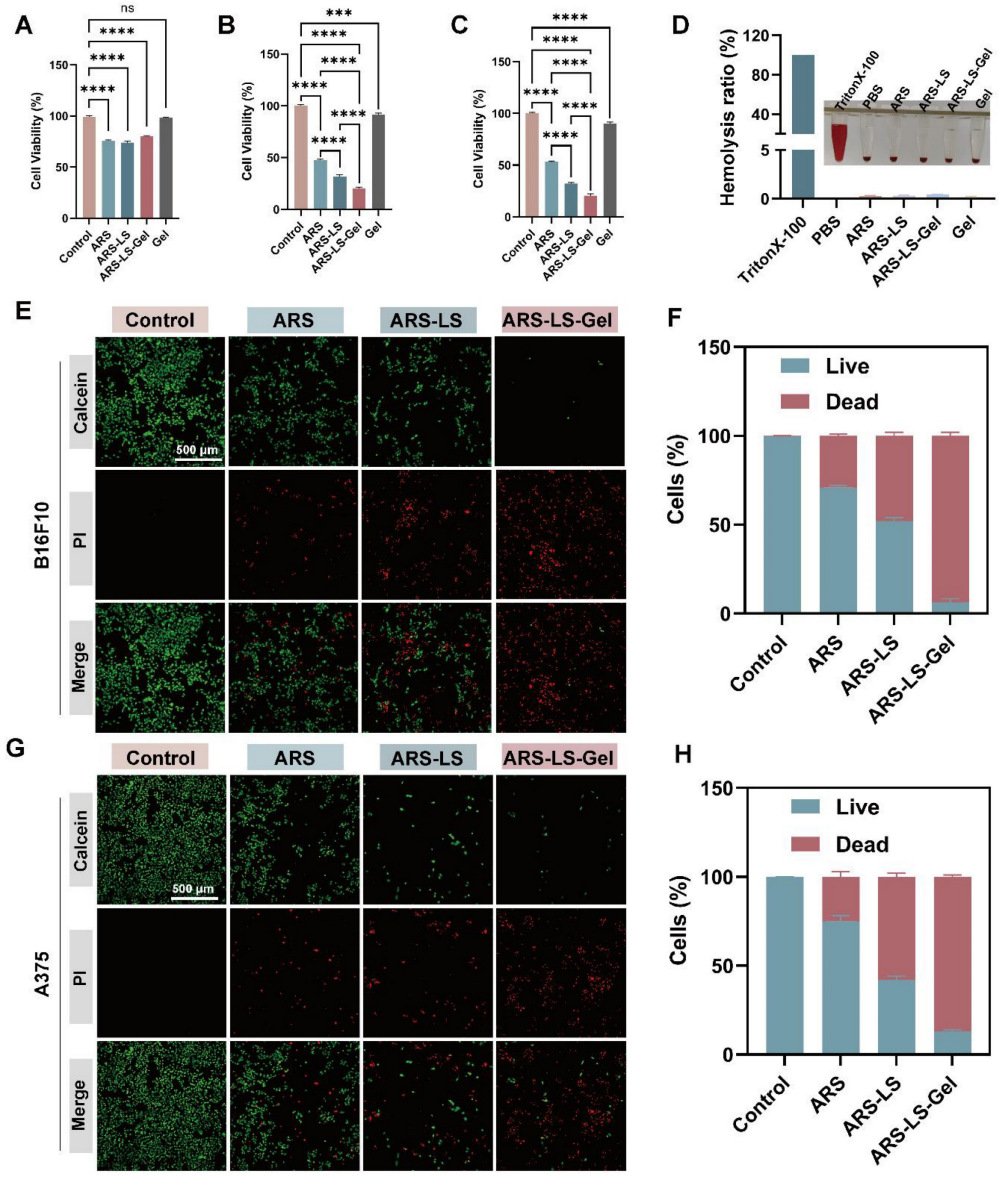
*In vitro* evaluation of ARS-LS-Gel biocompatibility and therapeutic efficacy. (A) Cytotoxicity assessment in L929 following treatment with ARS-LS-Gel and its components. (B-C) Antiproliferative effects in (B) B16F10 and (C) A375 melanoma cell lines. (D) Hemocompatibility analysis showing hemolysis rates of different formulations. (E-H) Therapeutic efficacy evaluation: (E) representative live/dead staining images (scale bar: 500 μm) and (F) corresponding quantification for B16F10 cells; (G) live/dead staining images (scale bar: 500 μm) and (H) quantification for A375 cells following various treatments. The data are presented as the means ± SDs (n = 3). ns (not significant), *P < 0.05, **P < 0.01, ***P < 0.001, ****P < 0.0001.

The optimal concentrations of 40 μM for B16F10 cells and 10 μM for A375 cells were determined through systematic Cell Counting Kit-8 (CCK-8) viability assays (Figure S4). The CCK-8 assay revealed excellent cytocompatibility of the blank hydrogel, with minimal cytotoxicity observed in NCTC clone 929 (L929) cells, incorporation of ARS-LS and ARS-LS-Gel, reducing L929 cell viability to 80.2% and 74.0%, respectively (Figure 3A). Notably, while the blank hydrogel exhibited only marginal tumor suppression in mouse melanoma cells (B16F10) and human melanoma cells (A375), ARS-LS-Gel treatment markedly enhanced therapeutic efficacy, decreasing viability to 20.33% (B16F10 cells) and 20.66% (A375 cells) (Figure 3B-C). This represented a statistically significant improvement over free ARS treatment (47.39% and 53.07% viability for B16F10 and A375 cells, respectively), confirming the superior antitumor performance of our nanoplatform.

Hemocompatibility assessment further supported the platform's biosafety, with all experimental groups demonstrating hemolysis rates below the 5% safety threshold (maximum 4.2%, Figure 3D) 54. These collective findings validate both the enhanced therapeutic efficacy and excellent biocompatibility of our ARS-LS-Gel system.

We further assessed the *in vitro* anticancer activity of ARS-LS-Gel using live/dead staining in B16F10 and A375 melanoma cell lines (Figure 3E-H). Fluorescence microscopy images showed viable cells (green) and dead cells (red), with ARS-LS and ARS-LS-Gel treatments demonstrating significantly enhanced cytotoxicity compared to both control and free ARS groups. This improved therapeutic effect can be attributed to the sustained drug release profile of our nanoformulations, which maintains effective intracellular drug concentrations over time.

### *In vitro* validation of the anti-tumor mechanisms of ARS-LS-Gel

#### Cellular uptake and ROS production

**Figure 4:**
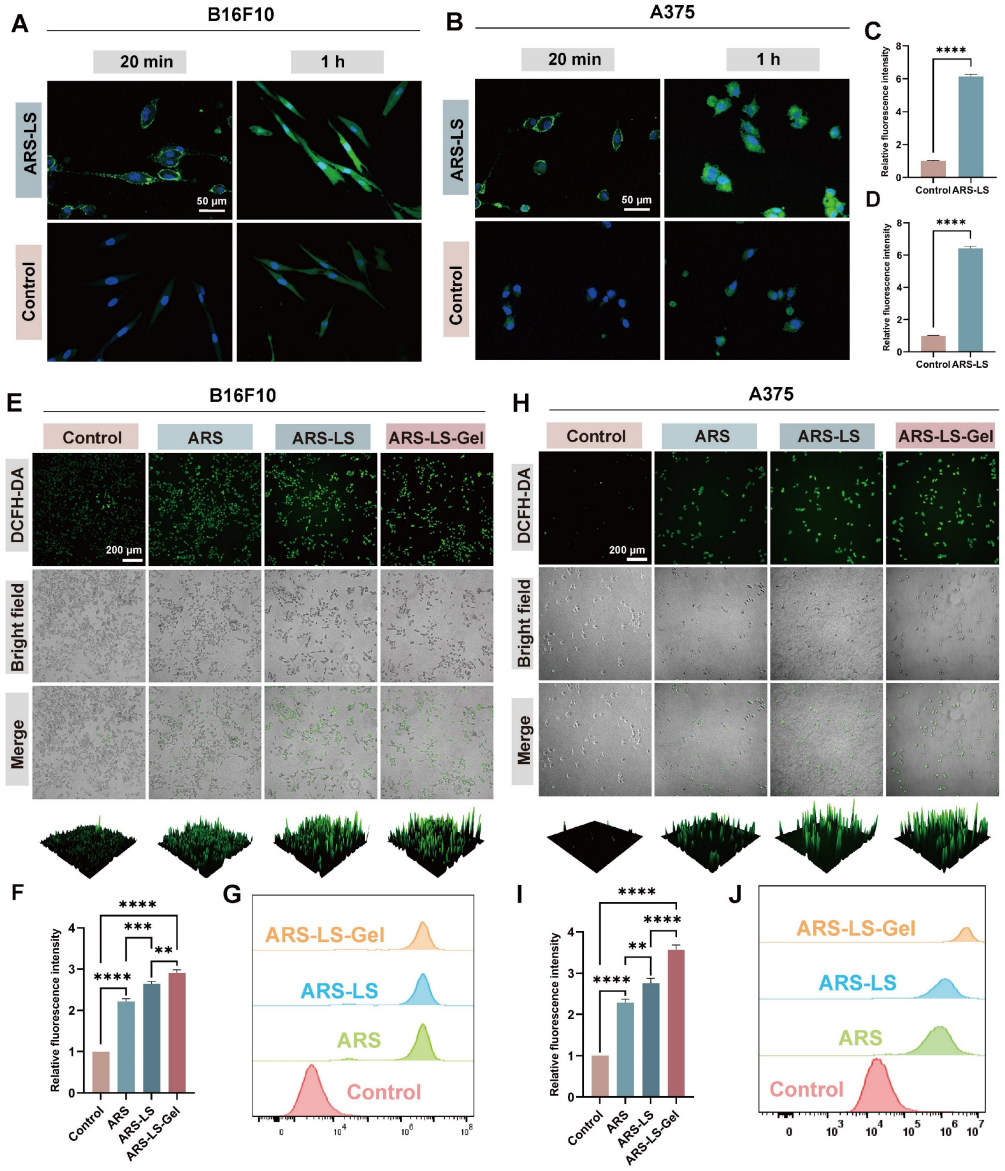
Cellular uptake and ROS production in melanoma cells following various treatments. (A-B) Cellular uptake visualization in (A) B16F10 and (B) A375 cells using fluorescence microscopy (scale bar: 50 μm) and quantitative analysis of relative fluorescence intensity in B16F10 (C) and A375 (D) after 1 h treatment. (E) Representative fluorescence images (scale bar: 200 μm), (F) corresponding quantitative analysis, and additional flow cytometry-based quantification (G) of DCFH-DA-stained B16F10 cells following different treatments. (H) Representative fluorescence images (scale bar: 200 μm), (I) corresponding quantitative analysis, and additional flow cytometry-based quantification (J) of DCFH-DA-stained A375 cells following different treatments. The data are presented as the means ± SDs (n = 3). ns (not significant), *P < 0.05, **P < 0.01, ***P < 0.001, ****P < 0.0001.

The therapeutic efficacy of nanomedicines is often contingent upon their cellular internalization 55. To evaluate this critical parameter, to comprehensively evaluate the cellular internalization process of ARS-LS particles, we performed confocal microscopy imaging of cells incubated with either FITC-labeled ARS-LS and FITC-labeled ARS for 20 min and 1 h (Figure 4A-B).

The results clearly demonstrated a time-dependent cellular entry pattern. After 20 min of incubation, FITC-labeled ARS-LS (green fluorescence) was primarily localized at the cell membrane, while after 1 h, substantial fluorescence was observed within the cytoplasm, indicating progressive cellular internalization. Statistical analysis revealed significant differences in fluorescence intensity between the ARS-LS and control groups at both time points (Figure 4C-D, S5), confirming that the liposomal formulation markedly enhances cellular uptake of ARS compared to the free drug.

Mitochondria serve as the primary source of intracellular reactive oxygen species (ROS). Under conditions of oxidative stress, mitochondrial dysfunction creates a vicious cycle of ROS overproduction 56. This redox imbalance initiates a cascade of apoptotic events, including dissipation of mitochondrial membrane potential (ΔΨm), cytochrome C release, and subsequent activation of the apoptotic pathway 57, 58. DCFH-DA staining and flow cytometry analysis revealed that ARS significantly elevated intracellular ROS levels in both B16 and A375 cells. Notably, the fluorescence intensity was markedly higher in the ARS-LS and ARS-LS-Gel groups compared to the control and ARS-only groups, suggesting that the sustained release of ARS from the drug delivery system enhances the intracellular accumulation of ROS (Figure 4E-J).

#### Mitochondrial dysfunction assessment

**Figure 5:**
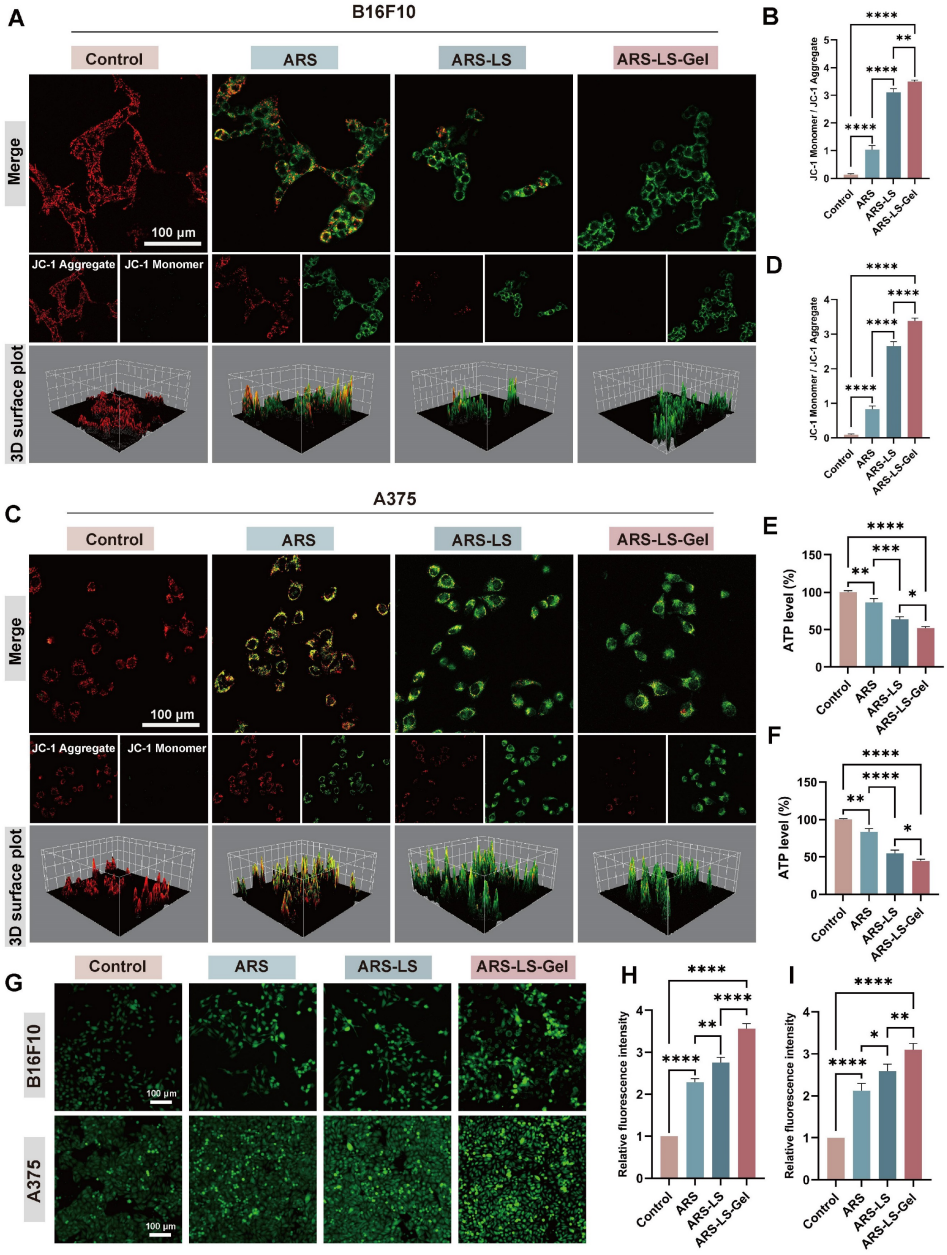
Mitochondrial dysfunction assessment showing ΔΨm alterations, ATP levels, and Ca²⁺ dysregulation in melanoma cells. (A) Fluorescence images (scale bar: 100 μm) and quantitative analysis (B) of mitochondrial membrane potential changes after various treatments of B16F10 cells. (C) Fluorescence images (scale bar: 100 μm) and quantitative analysis (D) of mitochondrial membrane potential changes after various treatments of A375 cells. (E-F) Quantitative analysis of ATP level in B16F10 and A375cells after treatment. Fluorescence images (G) (scale bar: 100 μm) and quantitative analysis (H-I) of Ca^2+^ in B16F10 and A375 cells after treatment. The data are presented as the means ± SDs (n = 3). ns (not significant), *P < 0.05, **P < 0.01, ***P < 0.001, ****P < 0.0001.

The collapse of ΔΨm represents a pivotal early event in apoptotic cascades, which can be quantitatively assessed using potentiometric fluorescent dyes, including JC-1 59, 60. We evaluated mitochondrial function by assessing ΔΨm using JC-1 staining in B16F10 and A375 cells and statistically analyzed the three samples (Figure 5A-D). JC-1 exhibits potential-dependent accumulation in mitochondria, forming red-fluorescent J-aggregates in polarized mitochondria, while remaining as green-fluorescent monomers in depolarized mitochondria. In the control cells (PBS-treated), there was a considerable number of red-emitting mitochondria. However, in the ARS-treated group, the number of red-emitting mitochondria was reduced, and very few red-emitting mitochondria were detected in the treated ARS-LS and ARS-LS-Gel groups, suggesting that ARS may have affected mitochondrial function and enhanced this effect in the presence of the nanoplatform.

Consistent with the observed mitochondrial membrane potential disruption, all treatment groups exhibited significantly reduced adenosine triphosphate (ATP) levels compared to controls, with the most pronounced decrease in the ARS-LS-Gel treatment group (39% reduction vs control in B16F10 cells, 51% reduction in A375 cells) (Figure 5E-F). This correlation reflects the central role of mitochondria as the primary ATP-producing organelles, where loss of membrane potential directly compromises oxidative phosphorylation capacity 61. The parallel reduction in both parameters confirms substantial mitochondrial dysfunction, as intact membrane potential is essential for maintaining the proton motive force required for ATP synthesis 62.

Notably, calcium ion (Ca²⁺) dysregulation further exacerbates mitochondrial impairment, serving as a critical regulator of tumor cell pathophysiology. Elevated intracellular Ca²⁺ concentrations disrupt normal calcium channel function, induce pathological Ca²⁺ overload, and directly compromise mitochondrial integrity 63. Quantitative assessment using the fluorescent Ca²⁺ indicator Fluo-4 AM revealed significantly enhanced intracellular Ca²⁺ levels in treated groups, with all formulations demonstrating markedly increased fluorescence intensity compared to controls, confirming successful induction of calcium overload (Figure 5G-I). The resulting cytosolic Ca²⁺ accumulation triggers a cascade of pathological events, including activation of Ca²⁺-dependent oxidases that amplify ROS production and inhibition of mitochondrial ATP synthesis through disruption of calcium-sensitive metabolic enzymes 64.

#### Cell apoptosis and mechanisms validation

**Figure 6:**
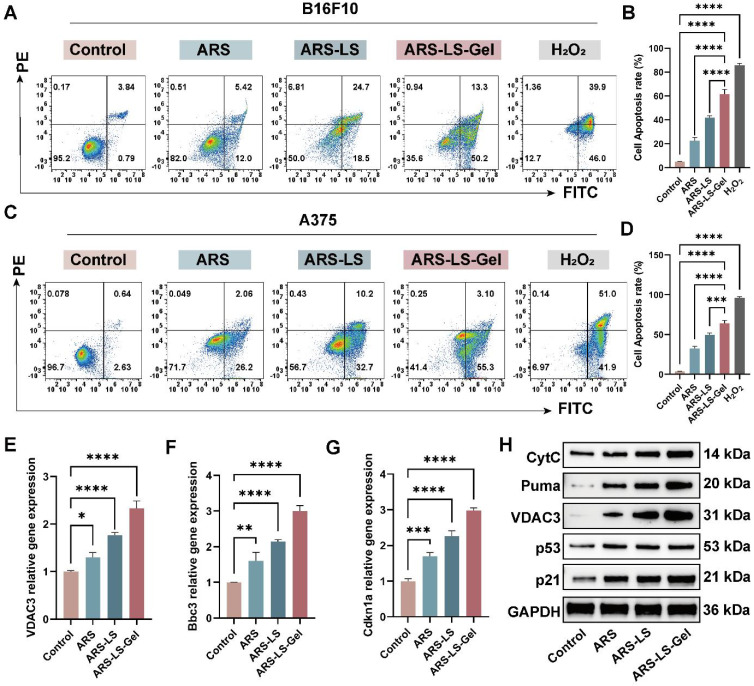
Apoptosis induction and its associated genes, protein expression in melanoma cells following treatment. (A) Representative flow cytometry dot plots showing Annexin V-FITC/PI staining of B16F10 cells after 48 h treatment. (B) Quantitative analysis of total apoptosis (early + late apoptotic populations) in B16F10 cells across treatment groups. (C) Corresponding flow cytometry profiles for A375 cells. (D) Quantification of apoptotic rates in A375 cells. (E-G) qPCR analysis of apoptosis-related gene expression: (E) VDAC3, (F) Bbc3 and (G) Cdkn1a mRNA levels. (H) WB analysis of apoptosis-related protein expression. The data are presented as the means ± SDs (n = 3). ns (not significant), *P < 0.05, **P < 0.01, ***P < 0.001, ****P < 0.0001.

To quantitatively assess treatment-induced apoptosis in melanoma cells, we performed flow cytometric analysis using Annexin V-FITC/propidium iodide (PI) dual staining. B16F10 and A375 cells were treated with various formulations for 24 (Figure S6) and 48 h, with hydrogen peroxide (H2O2, 50 μM) included as a positive control. To ensure analytical consistency across all flow cytometry data, we implemented a standardized gating strategy (Figure S7). The results showed that after 48-hour treatment, the apoptotic rate of B16F10 cells reached 63.50% in the ARS-LS-Gel group, significantly higher than those in the free ARS group (17.42%), control group (4.63%), and ARS-LS group (43.20%) (Figure 6A-B). In A375 cells (Figure 6C-D), the percentage of apoptosis was 58.40% after ARS-LS-Gel treatment for 48 h, which was significantly higher than that of the control (3.27%), free ARS (28.26%), and ARS-LS group (42.90%). At the same concentration, ARS-LS-Gel induced a 3.65-fold and 2.07-fold increase in the total apoptosis rate in B16F10 cells and A375 cells, respectively, compared to the free ARS group. These results emphasize that effective delivery of ARS can drive apoptosis in B16F10 and A375 cells.

To determine whether p53 signaling pathway-mediated mitochondrial dysfunction represents a key mechanism underlying ARS's anti-melanoma effects, we performed molecular validation using Quantitative real-time polymerase chain reaction (qPCR) and Western blot (WB) analysis. Our results demonstrated significant upregulation of key transcripts: Bbc3, Cdkn1a, and VDAC3 (Figure 6E-G), apoptotic proteins: Puma (Bbc3), p21 (Cdkn1a), VDAC3, CytC, and p53 (Figure 6H). These findings collectively reveal that ARS exerts its therapeutic effects through: activation of the p53 signaling pathway, induction of mitochondrial dysfunction, and promotion of apoptotic cascades.

### *In vivo* anti-tumor evaluation

**Figure 7:**
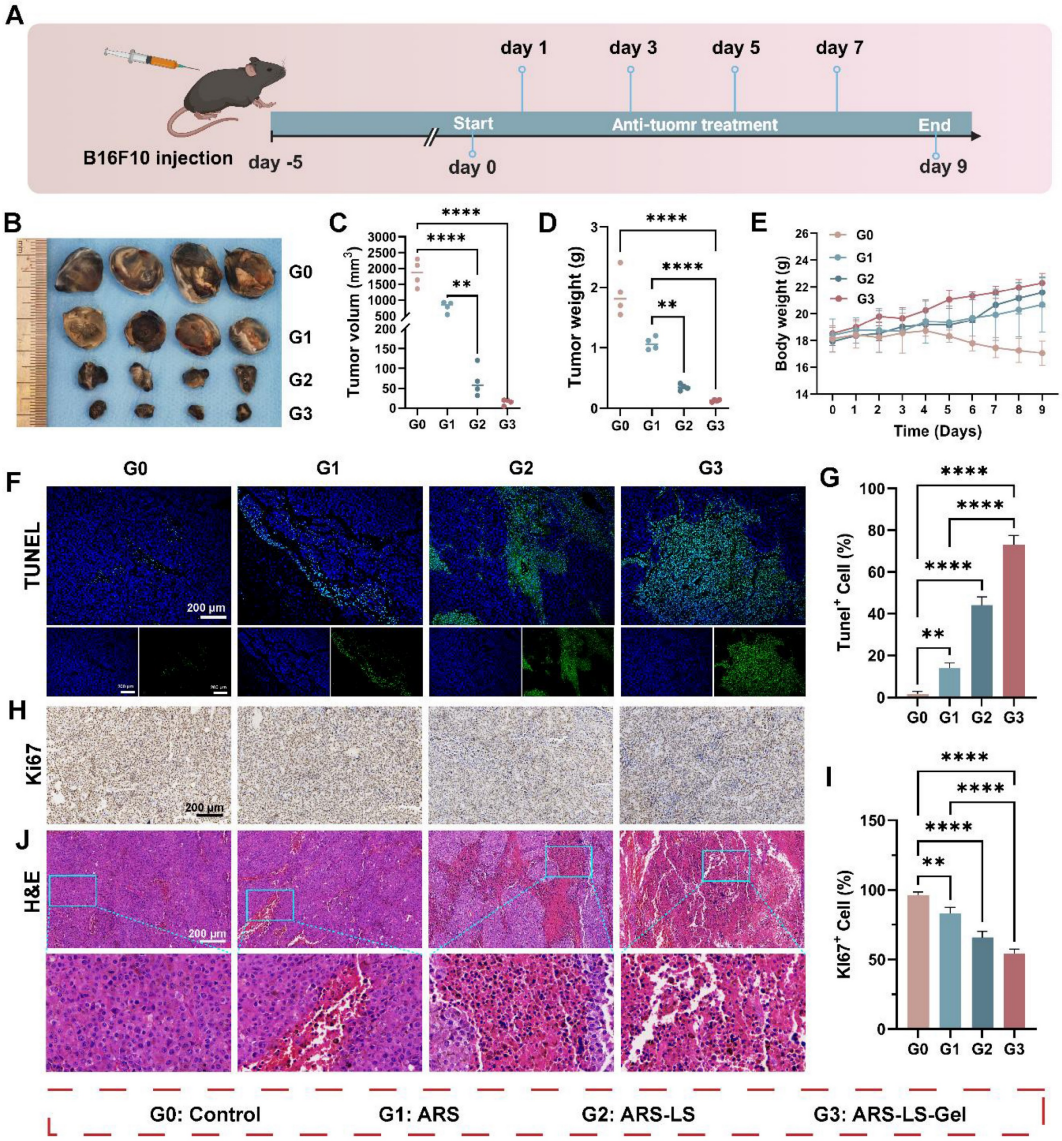
*In vivo* therapeutic evaluation of ARS formulations in a murine melanoma model. (A) Experimental design schematic showing treatment regimen and monitoring timeline. (B) Representative photographs of excised tumors collected on day 9 post-treatment. (C-D) Quantitative assessment of (C) tumor volume and (D) tumor weight across treatment groups at the study endpoint, data presented as mean ± SD (n = 4). (E) Longitudinal monitoring of body weight changes in different treatment groups throughout the experimental period, data presented as mean ± SD (n = 4). (F-G) Apoptosis evaluation showing (F) representative fluorescence images (scale bar: 200 μm) and (G) quantitative analysis of apoptotic cells following various treatments, data presented as mean ± SD (n = 3). (H-I) Proliferation assessment demonstrating (H) immunohistochemical staining (scale bar: 200 μm) and (I) quantitative analysis of Ki67-positive cells in treated tumors, data presented as mean ± SD (n = 3). (J) Histopathological examination of tumor tissues by H&E staining (scale bar: 200 μm). The data are presented as the means ± SDs (n = 3 or 4). ns (not significant), *P < 0.05, **P < 0.01, ***P < 0.001, ****P < 0.0001.

Building upon the demonstrated *in vitro* anti-melanoma efficacy of ARS-LS-Gel, we evaluated its therapeutic potential *in vivo* using a syngeneic melanoma mouse model (B16F10-bearing C57BL/6 mice). Figure 7A illustrates the treatment protocol. The results show that free ARS exhibited significant tumor-suppressive effects compared to the control group, with ARS-LS and ARS-LS-Gel demonstrating even greater therapeutic efficacy, markedly delaying tumor progression (Figure 7B). Quantitative analysis demonstrated free ARS showed significant anti-tumor activity, and the nanoplatform-enhanced formulations exhibited superior efficacy (Figure 7C-D). Body weight monitoring revealed that the ARS-LS-Gel treatment group exhibited the highest weight gain; control mice experienced a gradual decline in body weight nine days post-inoculation (Figure 7E), likely due to cachexia in the absence of effective treatment 65.

To assess histological changes and tumor-related protein expression, we performed hematoxylin and eosin staining (H&E) and immunofluorescence staining on B16F10 tumor tissues. Terminal deoxynucleotidyl transferase dUTP nick end labeling (TUNEL) staining and quantitative analysis of TUNEL staining confirmed ARS-induced apoptosis *in vivo*, with ARS-LS and ARS-LS-Gel treatments yielding substantially higher apoptotic cell counts (Figure 7F-G). Ki-67 immunostaining revealed that ARS significantly suppressed tumor cell proliferation, with the ARS-LS-Gel group showing minimal Ki-67-positive cells (brown-yellow staining), indicating potent anti-proliferative effects (Figure 7H). Quantitative analysis of Ki-67 revealed potent proliferation inhibition, with ARS-LS-Gel treated tumors exhibiting only 54.3 ± 2.1% Ki-67^+^ cells compared to 96.3 ± 1.7% in controls (Figure 7I). H&E staining further demonstrated pronounced morphological alterations in ARS-treated tissues (particularly ARS-LS and ARS-LS-Gel), including cell shrinkage, nuclear condensation, and fragmentation—hallmarks of apoptosis 66 —along with a notable reduction in mitotic figures, consistent with inhibited melanoma growth (Figure 7J).

### *In vivo* wound healing efficacy

**Figure 8:**
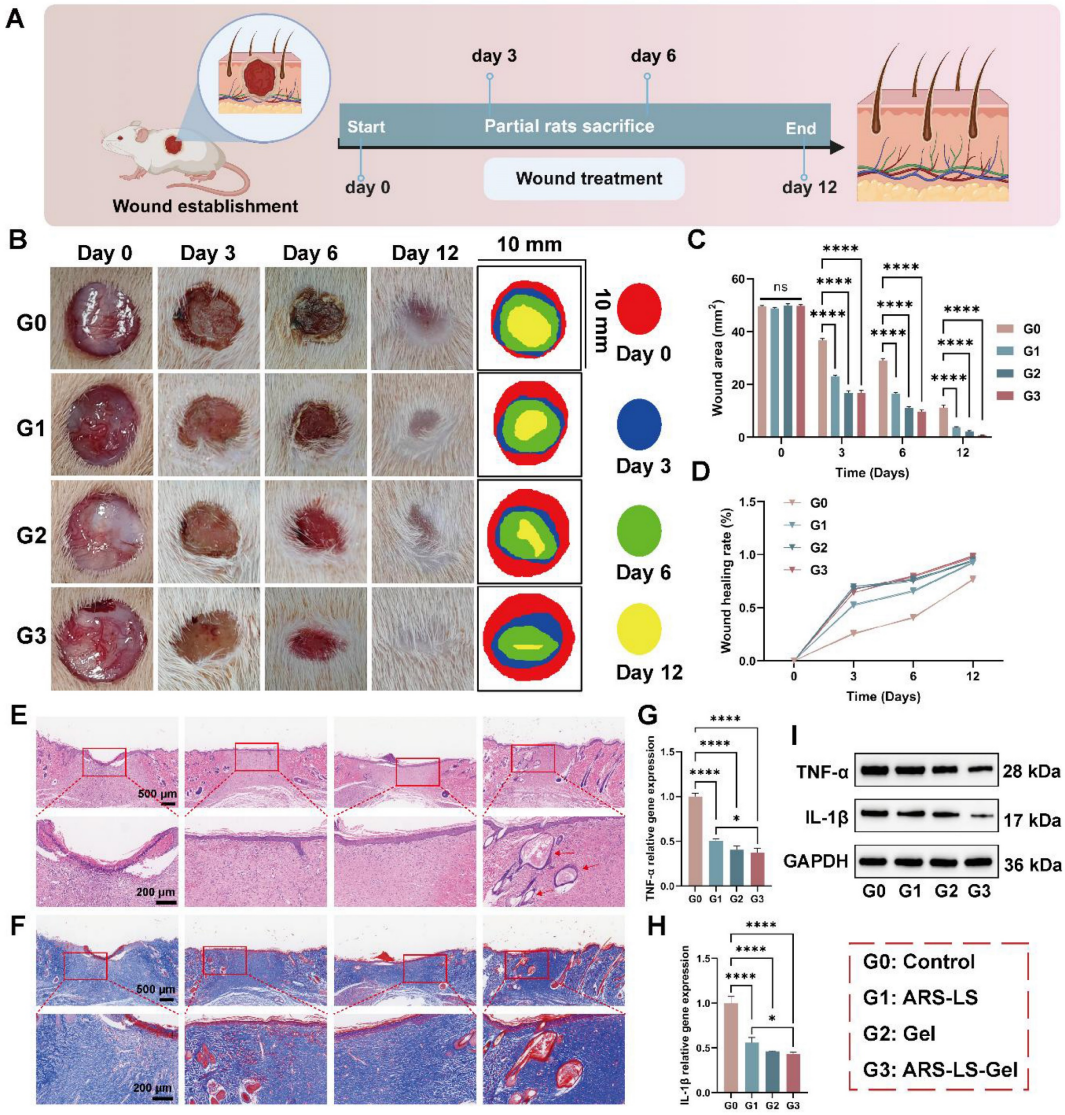
Wound healing evaluation in SD rats full-thickness skin defect model. (A) Experimental design schematic showing treatment protocol and monitoring timeline. (B) Representative macroscopic images documenting wound appearance and contraction patterns throughout the study period. (C-D) Quantitative assessment of (C) absolute wound area and (D) healing rate progression across treatment groups. (E) Histopathological evaluation by H&E staining at day 12, demonstrating epidermal regeneration and inflammatory responses (scale bar: 200 μm). (F) Collagen deposition analysis via Masson's trichrome staining at day 12, revealing extracellular matrix reorganization. (G-H) qPCR analysis of pro-inflammatory cytokine expression. (G) TNF-α and (H) IL-1β mRNA levels in wound tissues. (I) TNF-α and IL-1β protein levels in wound tissues. The data are presented as the means ± SDs (n = 3). ns (not significant), *P < 0.05, **P < 0.01, ***P < 0.001, ****P < 0.0001.

Rapid and effective wound protection and closure are crucial for preventing infections and alleviating pain 67, we systematically evaluated cutaneous regeneration using a full-thickness excisional wound model in Sprague-Dawley (SD) rats. Figure 8A illustrates the experimental procedure for rat skin treatment. By day 12, all groups exhibited wound contraction, with the ARS-LS and ARS-LS-Gel groups demonstrating superior wound closure compared to the control group. Notably, the ARS-LS-Gel group achieved the smallest relative wound area at this time point (Figure 8B-C). Quantitative analysis of wound closure kinetics revealed significantly accelerated healing rates across all treatment groups compared to controls. Specifically, the ARS-LS-Gel group demonstrated the most pronounced effect, achieving 98 ± 0.21% wound closure by day 12 post-treatment versus 77 ± 1.38% in the control group, suggesting that treatment with ARS-LS-Gel significantly accelerated wound closure (Figure 8D).

Histological evaluation via H&E staining revealed distinct patterns of skin repair. While the control group maintained a pronounced inflammatory response at day 12, the ARS group showed attenuated inflammation, as evidenced by reduced inflammatory cell infiltration. The ARS-LS and ARS-LS-Gel groups displayed enhanced epidermal regeneration, with the ARS-LS-Gel group additionally exhibiting neoformation of skin appendages, including hair follicles (indicated by red arrows, Figure 8E). Masson's trichrome staining demonstrated improved collagen deposition in all treatment groups relative to controls, with more abundant and tightly organized collagen fibers, indicative of advanced tissue remodeling (Figure 8F). This finding aligns with established wound healing paradigms, where controlled inflammation and robust collagen synthesis are critical for successful tissue regeneration 68-71.

The anti-inflammatory properties of the treatments were further confirmed by qPCR and WB analysis, which revealed significant downregulation of pro-inflammatory cytokines TNF-α and IL-1β (Figure 8G-I). This suppression of inflammatory mediators, attributable to the combined effects of ARS and carboxymethyl chitosan 72, 73, likely contributed to the observed therapeutic outcomes.

### Comprehensive assessment of biocompatibility

Histopathological examination of major organs (heart, liver, spleen, lungs, and kidneys) in both C57/BL6 mice and SD rats revealed no abnormalities following treatment with ARS-LS-Gel. Furthermore, subcutaneous implantation of ARS, ARS-LS-Gel and ARS-LS-Gel in mice induced no significant alterations in complete blood counts (red blood cells, white blood cells, hemoglobin, hematocrit, platelets) or serum biochemistry (alanine, aminotransferase, aspartate aminotransferase, blood urea nitrogen, blood urea nitrogen, creatinine) after 72 h, with all parameters remaining within normal physiological ranges (Figure S8). These collective findings demonstrate the excellent *in vivo* biosafety profile of ARS-LS-Gel, supporting its potential as a promising nanoplatform for postoperative melanoma combination therapy.

To characterize its biodegradation behavior, we first evaluated the *in vitro* degradation of blank hydrogel and ARS-LS-Gel in PBS (pH 7.0), observing > 85% mass loss within 5 days (Figure S9). Subsequent *in vivo* studies showed that a 0.1 mL subcutaneous implant in mice degraded completely within 12 days (Figure S10), correlating with its therapeutic timelines (12-day wound healing and 9-day tumor suppression). This synchronous degradation-release profile, along with the absence of toxicity, confirms ARS-LS-Gel's potential as a safe, transient therapeutic platform.

## Conclusion

The dual challenge of preventing melanoma recurrence while promoting post-surgical wound healing remains a critical unmet need in oncology. Building on its known pharmacological properties, we hypothesized that artesunate (ARS) possesses significant anti-melanoma activity, a premise subsequently confirmed through transcriptomic profiling. To address this dual therapeutic need, we developed an artesunate nanoplatform (ARS-LS-Gel) designed to simultaneously inhibit tumor growth and accelerate wound healing, with its mechanism systematically investigated. Mechanistic studies revealed that ARS-LS-Gel induced apoptosis in B16F10 cells, thereby suppressing melanoma proliferation. The platform triggered mitochondrial dysfunction via p53 pathway activation, leading to ROS accumulation and cytochrome C release - a mechanism initially identified through transcriptomic analyses, including GO enrichment and clustering. In addition, a range of manifestations of mitochondrial dysfunction, such as decreased mitochondrial membrane potential, decreased ATP production, and calcium overload, have been observed. Consistent results were observed in A375 cells *in vitro*, supporting the potential translational relevance to human applications. *In vivo* experiments demonstrated that ARS-LS-Gel effectively suppressed melanoma progression while enhancing cutaneous wound repair through reducing inflammation and promoting collagen fibrillation in a rodent model. Collectively, these findings establish ARS-LS-Gel as a promising dual-function therapy for post-surgical melanoma management, addressing both tumor recurrence prevention and wound regeneration, while offering a potential solution to the challenge of increasing drug resistance in melanoma treatment.

## Methods

### Preparation of ARS-LS-Gel

Soy lecithin (L861761, Macklin, China) and cholesterol (C804517, Macklin, China) were dissolved in anhydrous ethanol at a mass ratio of 10:5:1 (soylecithin:cholesterol:ethanol). Artesunate (ARS; A800614, Macklin, China) was subsequently added to the organic phase and completely dissolved. The solution was rotary evaporated at 40 °C (Rotavapor R-300, Switzerland) to form a uniform lipid film on the flask walls. The film was then hydrated with PBS, followed by 5-minute probe sonication (VCX 750, USA) at 40% amplitude. The resulting suspension was sequentially extruded through 0.22 μm polycarbonate membranes (Jet Biofil, China) 20 times to obtain monodisperse artesunate-loaded liposomes (ARS-LS). Carboxymethyl chitosan (CMCS, C902396, Macklin, China) and carbomer 940 (CBM940, C832684, Macklin, China) were dissolved in deionized water, and then the solutions were mixed under constant stirring to give final concentrations of 0.025% (w/v) and 0.5% (w/v), respectively. The solutions were then combined under constant stirring to ensure homogeneous mixing. The pH was adjusted to 7.0 ± 0.2 using triethanolamine, resulting in the formation of a blank hydrogel matrix. For ARS-LS-Gel preparation, ARS-loaded liposomes (ARS-LS) were incorporated into the hydrogel base using the same procedure, with the liposome suspension added during the mixing phase to ensure uniform distribution. Regarding formulation preparation, the ARS content in both ARS-LS and ARS-LS-Gel was strictly equivalent to that of free ARS. This was quantitatively determined by measuring the weight difference between freeze-dried blank liposomes versus ARS-LS, as well as blank hydrogel versus ARS-LS-Gel, thereby ensuring dose comparability between the two formulations.

### Characterization of ARS-LS-Gel

Malvern Zetasizer Nano ZS (Nano ZS, UK) was used for determining the particle size, zeta potential, and PDI of liposomes at 25 °C. Data analysis was based on the average value of three parallel tests. The particle size, zeta potential, and PDI of the ARS-LS were determined at 0, 7, 14, 28, and 42 days after sample preparation. These measurements were used to evaluate the stability of the sample stored at 4 °C. ARS-LS were diluted twice with purified water, and an appropriate amount of liposome diluent was dropped onto a copper mesh. After the copper mesh was dried at room temperature, the ARS-LS were photographed by Transmission Electron Microscopy (TEM, FEI Tecani G2, USA). The ARS-LS-Gel samples were frozen at -20 °C for 24 h and subsequently lyophilized using a vacuum freeze-dryer (LABCONCO, USA) for 24 h. For cross-sectional analysis, samples were carefully sectioned using a surgical scalpel and mounted on aluminum stubs. The exposed surfaces were sputter-coated with a 10 nm gold layer to enhance conductivity. Morphological examination was performed using field-emission scanning electron microscopy (SEM, Inspect F50, Thermo Fisher, USA) at an accelerating voltage of 5 kV under high vacuum conditions.

### FTIR test

Fourier transform infrared spectroscopy (FTIR, Beifen-Ruili, China) was used to analyze the chemical structures of ARS-LS-Gel. The spectral range was configured as 400-4000cm^-1^, and the mode was set to transmittance. The data were imported into Origin for analysis.

### Drug release

A certain mass of ARS was dissolved in dimethyl sulfoxide (DMSO) and added to PBS buffer to form a series of solutions of different concentrations, and the absorbance at 210 nm was measured by a UV spectrophotometer (UV3600plus, USA) to obtain the standard curve. ARS-LS and ARS-LS-Gel were individually sealed in dialysis bags (MWCO 8000-14000 kDa, H1562, Haoma, China) and immersed in 20 mL of release medium (PBS at pH 7.0 and 5.4, simulating physiological and acidic microenvironments, respectively), and drug release experiments were performed at 37 ℃ on a shaker at 100 rpm. 2 mL samples were taken at 0, 2, 4, 6, 8, 10, 12, 24, 48, 72 and 96 h, respectively. The ARS was quantified by UV spectrophotometry, and the cumulative release of ARS at different times was calculated according to the standard curve equation.

### The loading content (LC) and encapsulation efficiency (EE) test

The absorbance method was used to determine the loading (LC) and encapsulation efficiency (EE) of ARS in ARS-LS and ARS-LS-Gel.

The formula for calculating LC and EE is as follows:

LC (%) = (W0 - CsV)/W1 × 100%

EE (%) = (W0 - CsV)/W0 × 100%

W0 is the initial total weight of ARS, Cs is the concentration of ARS in the supernatant after loading, V is the volume of the supernatant after loading, and W1 is the total weight of ARS-LS and ARS-LS-Gel.

### Cell culture

Mouse melanoma cells (B16F10) (RRID: CVCL_0159), human melanoma cells (A375) (RRID: CVCL_0132) and NCTC clone 929 (L929) (RRID: CVCL_0462) were purchased from Biotechnology Company (Guangzhou Kefan, China), and all cell lines were rigorously authenticated and confirmed to be free of contamination through comprehensive Short Tandem Repeat (STR) profiling. All types of cells were cultured in a highly glycosylated DMEM (C11995500BT, Gibco, USA) medium containing 10% fetal bovine serum (FBS, P1020-500, Solarbio, China) and 1% streptomycin/penicillin (15140122, Gibco, USA). The culture environment was set at 37 °C and 5% CO_2_.

### CCK-8

The optimal concentrations of 40 μM for B16F10 cells and 10 μM for A375 cells were determined through systematic CCK-8 viability assays. For B16F10 cells and normal murine L929 fibroblasts, ARS was dissolved in DMSO (final concentration < 0.1%) and tested at four concentrations (10, 20, 40, and 80 μM) over 72 h. Parallel experiments using A375 cells and human umbilical vein endothelial cells (HUVECs) evaluated four lower concentrations (2.5, 5, 10, and 20 μM) under identical conditions. The selection criteria required: 60% inhibition of tumor cell proliferation and < 30% cytotoxicity to normal counterparts. These thresholds ensured therapeutic efficacy while maintaining safety, with 40 μM and 10 μM demonstrating optimal selectivity for B16F10 and A375 cells, respectively (Figure S4). For *in vitro* evaluations, these cell-type-specific concentrations were then rigorously maintained across all subsequent mechanistic studies (live/dead cell assays, apoptosis analysis, ROS detection, mitochondrial membrane potential assessment, etc.).

B16F10 cells, L929 cells, HUVECs cells, and A375 cells (5000 cells per well) were inoculated in 96-well plates. Then, Free ARS (40 μM for B16F10/L929; 10 μM for A375), ARS-LS (ARS-equivalent dose), and ARS-LS-Gel (ARS-equivalent dose) were added and incubated for 24, 48, and 72 h. CCK8 solution was added for 1 h. Finally, it was then measured at 450 nm using an enzyme marker (SynergyH1, USA).

### Hemolysis assay

Mouse erythrocytes were resuspended in 5% (v/v) PBS and incubated with individual samples for half an hour at 37 °C. Sterile PBS and 0.1% Triton X-100 were used as negative and positive controls, respectively. All samples were centrifuged, and 100 μL of supernatant was transferred to a 96-well plate, and the absorbance was measured at 540 nm using an enzyme marker (SynergyH1, USA). The hemolysis rate was calculated as (absorbance [test] - absorbance [negative])/(absorbance [positive] - absorbance [negative]) *100%.

### Live/Dead assay

B16F10 cells and A375 cells were cultured in 12-well plates and treated with PBS, ARS, ARS-LS, and ARS-LS-Gel, respectively, for 72h. The plates were washed 3 times with PBS. Serum-free medium containing Live/dead stain (CA1630-500T, Solarbio, China) was added, incubated at 37 °C, 5% CO_2_ for 20 min. Observe under an inverted fluorescence microscope (Leica, Wetzlar, Germany) after incubation.

### Cellular uptake

B16F10 and A375 cells were inoculated in Confocal Petri dishes (1× 10^5^ cells/well), incubated with FITC-marked ARS-LS or FITC-marked ARS for 20 min and 1 h, then washed with PBS. Cellular uptake in each group was observed using a confocal laser scanning microscope (Leica, Germany).

### RNA-seq and data analysis

Total RNA was extracted from B16F10 cells using Trizol reagent (YZ-15596018, Solarbio, China). Sequencing was performed with the Illumina Novaseq 6000 / MGISEQ-T7 sequencing platform. Differential expression analysis of genes between groups was performed using DESeq2 with default screening thresholds for differentially expressed genes of | log2FC | > 1 and P.adj < 0.05. Gene Ontology (GO) and Kyoto Encyclopedia of Genes and Genomes (KEGG) enrichment analyses were performed on the differential genes. Function enrichment and KEGG pathway enrichment analyses were performed using the cluster Profiler R package, and when P < 0.05, this GO or KEGG function was considered significantly enriched.

### Intracellular ROS generation

B16F10 cells and A375 cells (2 × 10^5^ cells per well) were inoculated in a 6-well plate. The cells were treated with PBS, ARS, ARS-LS, and ARS-LS-Gel for 24 h and then incubated with DCFH-DA (S0033S, Beyotime, China) for 30 min. Finally, the cells were photographed by a fluorescence imaging system (Olympus IX73, Japan) and quantified by flow cytometry.

### Determination of mitochondrial membrane potential (MMP, Δψm)

B16-F10 cells and A375 cells were cultured in a Confocal Petri dish (BS-15-GJM, biosharp, China) at a density of 1×10^5^ cells/well for 24 h and then treated with PBS, ARS, ARS-LS, and ARS-LS-Gel for 24 h. The mitochondrial membrane potential was then detected with a JC-1 Kit (C2003S, Beyotime, China); first, cells were washed twice in PBS, and the medium was replaced with serum-free medium. Then, the cells were stained with JC-1 dye at 37 °C for 20 min in accordance with the manufacturer's instructions.

### ATP quantification assay

B16F10 and A375 cells were treated with PBS, ARS, ARS-LS, and ARS-LS-Gel. The cellular ATP levels were quantified using the ATP Assay Kit (S0026, Beyotime, China) according to the manufacturer's protocol. Briefly, cells were lysed in the provided extraction buffer and centrifuged. The supernatant was transferred to a 96-well white microplate and mixed with the reaction solution. Chemiluminescence was measured using a multimode microplate reader (SynergyH1, USA) at 340nm. ATP concentrations were calculated against a standard curve.

### Measurement of Ca^2+^ ion levels

B16F10 and A375 cells were seeded in 12-well plates at a density of 1×10^5^ cells/well and allowed to adhere for 24 h. Following treatment with PBS, ARS, ARS-LS, and ARS-LS-Gel, cells were loaded with Fluo-4 AM calcium indicator (F8500, Solarbio, China) for 15 min at 37 ℃ in serum-free medium. After three washes with PBS, intracellular calcium levels were immediately quantified using an inverted fluorescence microscope (Olympus, Japan).

### Cell apoptosis

Apoptosis was explored through Annexin V-FITC-PI staining (C1062M, Beyotime, China) followed by flow cytometry analysis. Specifically, B16F10 cells and A375 cells were subjected to PBS, ARS, ARS-LS, and ARS-LS-Gel for 24 and 48 h, and then stained with PI and Annexin V-FITC according to the manufacturer's instructions. Subsequently, analysis was conducted utilizing a CytoFlex S flow cytometer (Muse^TM^, USA).

### qPCR

Total RNA was extracted from B16F10 cells and rat wound tissues using the RNA Extraction Kit (Invitrogen, Carlsbad, CA, 15596-026) following the manufacturer's protocol. RNA quality was verified by electrophoresis and spectrophotometric analysis prior to cDNA synthesis using the PrimeScript RT Reagent Kit (RR047A, Takara, Japan) in 20 μL reaction volumes. Quantitative real-time PCR analysis was performed to evaluate expression profiles of apoptosis-related genes (Bbc3, Cdkn1a, VDAC3) in melanoma cells and inflammatory cytokines (TNF-α, IL-1β) in wound tissues, with GAPDH serving as the housekeeping gene. The primer sequences are shown in Table S1.

### Western blot

The total protein concentration was measured using the BCA kit (P0012, Beyotime, China). Subsequently, equal quantities of proteins were subjected to SDS-polyacrylamide gel electrophoresis and subsequently transferred to polyvinylidene fluoride membranes (PVDF, IPVH00010, Millipore, USA). Following incubation with 5% skimmed milk, the membranes were incubated overnight at 4 ℃ with the primary antibodies: BBC3 (1:1000, ab9643, Abcam, USA), VDAC3 (1:1000, ab14734, Abcam, UK), P53 (1:500, ab240, Abcam, UK), CytC (1:1000, K009448P, Solarbio, China), Cdkn1 (1:1000, ab102013, Abcam, UK), TNF-α (1:500, ab215188, Abcam, UK), IL-1β (1:1000, ab234437, Abcam, UK) and GAPDH (1:200, ab8245, Abcam, UK). Following washing with Tris-HCl buffered saline and Tween (TBST, T917681, Macklin, China), the membranes were incubated for 1 h at room temperature with the second antibody (1:5000, ab288151, Abcam, UK) for 1 h at room temperature. Following a subsequent wash with TBST, the mixed ECL luminescent solution was applied to the front side of the membrane. Protein signals were identified utilizing a fully automated chemiluminescence analyzer (Tanon-5200, China).

### Animals breeding

C57BL/6 mice (male, 6 to 8 weeks old) and Sprague-Dawley rats (SD rats) (male, 8 weeks old) were obtained from Southwest Medical University Laboratory Animal Center. The mice and rats were kept under SPF conditions at the Animal Laboratory Center of Southwest Medical University. All animal experiments adhered to ethical standards and received ethical certification (SWMU20250085).

### *In vivo* antitumor efficacy

A tumor-bearing mouse model was constructed by injecting B16F10 cells (2 × 10^6^ cells / 100 μL) subcutaneously into the right axilla of C57BL/6 mice. When the tumor volume reached ~ 50 mm³ (Figure S11), the mice were randomly divided into 4 groups (n = 4 per group) and injected with PBS, ARS, ARS-LS, and ARS-LS-Gel through the local injection within the tumor on Days 0, 1, 3, 5, and 7. The changes in mouse weight were recorded during treatment. On Day 9, tumor tissue was obtained from the mice, photographed, and weighed. After the end of the anti-tumor efficacy experiment, the mice were sacrificed by euthanasia, and the hearts, livers, Spleen, Lungs, and kidneys of the mice were taken out. The organs were fixed with 10% formalin, embedded in paraffin, sectioned, stained with H&E, and the tumor tissues were detected with immunofluorescence and Immunohistochemistry evaluation.

### *In vivo* animal skin repair experiment

Skin repair experiment was carried out on SD rats (8-week-old, male, 200-220 g). After anesthetization, the dorsal hair of SD rats was shaved, and a 1.0 cm × 1.0cm skin defect was constructed on the back. The rats were randomized into 4 groups (n = 3 per group): Control group, blank hydrogel, ARS-LS, and ARS-LS-Gel. The wound healing study followed an extended alternate-day schedule (days 1, 3, 5, 7, 9, and 11 post-grouping). At days 0, 3, 6, and 12, the wound healing was photographed, and the wound areas were measured by Image J software. Subsequently, all rats were sacrificed in each group at day 12, and the whole wound sites (including the wound and the surrounding normal skin tissues) were excised and stained with H&E and Masson staining. qPCR and WB for the detection of TNF-α and IL-1β gene and protein levels in rat skin wound tissue.

### Statistical analysis

The experimental data are expressed as the mean ± SD. Two independent samples were compared using a t-test, and multiple samples were compared using one-way analysis of variance (ANOVA). A p-value <0.05 was considered statistically significant, with specific comparisons denoted as follows: ns (not significant), *P < 0.05, **P < 0.01, ***P < 0.001, ****P < 0.0001.

## Supplementary Material

Supplementary figures and table.
