# Supplementary material for: Artesunate nanoliposome-hydrogel: a dual-modal therapy for post-surgical melanoma
Source: Theranostics. 2026 Jan 1;16(2):580–98. doi: 10.7150/thno.120793 (PMC12674888; doi:10.7150/thno.120793)
Supplement: Supplementary file 1 — Supplementary figures and table. [file thnov16p0580s1.pdf]

Artesunate nanoliposome-hydrogel: a dual-modal therapy for post-surgical melanoma

Hongmei Chen[1, 2, 3], Zhongke Wang[1, 2, 3], Ying Huang[1, 2, 3], Aodi Li[1, 2, 3], Xinlei Xiong[1, 2, 3], Yifan Pu[1, 2, 3], Ling Guo[1, 2, 3,*]

[1] School of Stomatology, Southwest Medical University, Luzhou, China.
[2] Luzhou Key Laboratory of Oral & Maxillofacial Reconstruction and Regeneration, Luzhou, China.
[3] Department of Prosthodontics, The Affiliated Stomatological Hospital of Southwest Medical University, Luzhou, China.

[*]Corresponding author: E-mail: glsmiling@swmu.edu.cn.

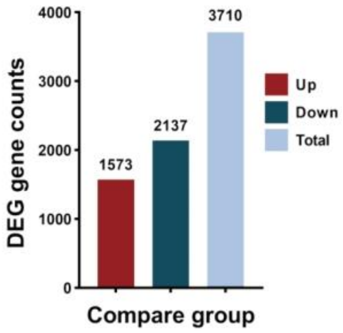

Figure S1. The RNA-seq dataset revealed a total of 3710 (1573 up, 2137 down) DEGs.

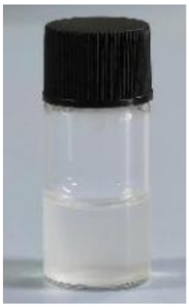

Figure S2. Appearance of ARS-LS.

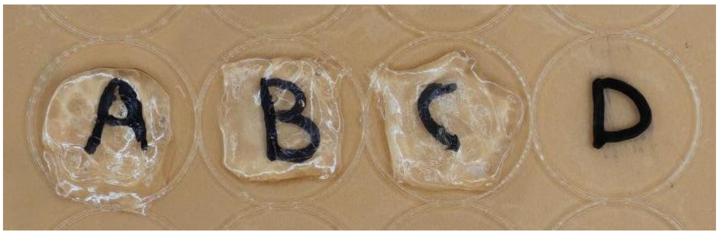

Figure S3. Transparency test of ARS-LS-Gel.

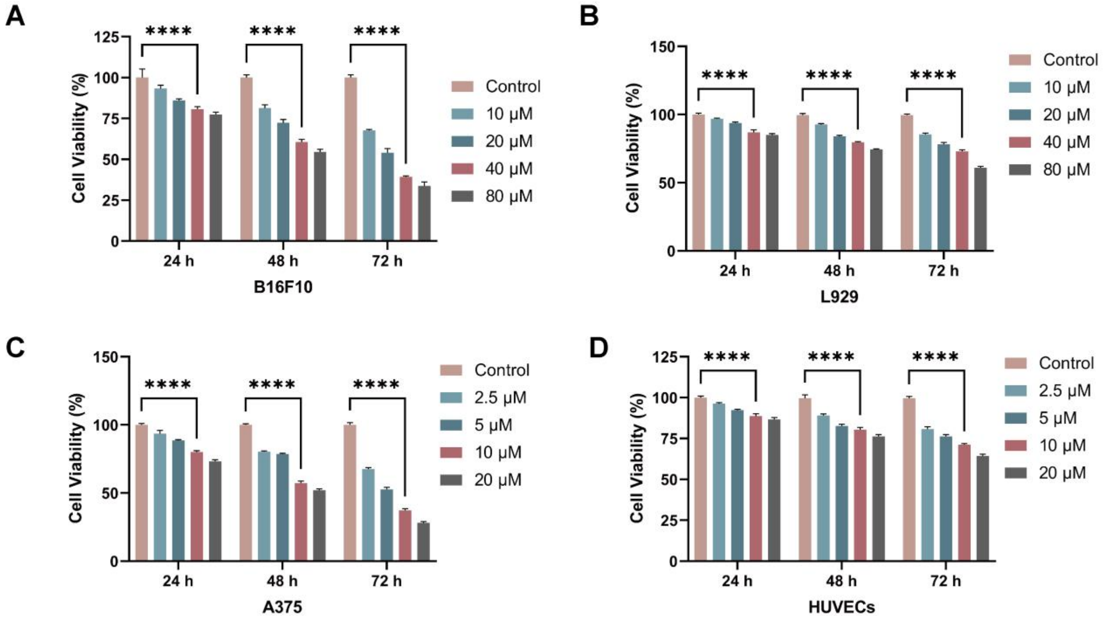

Figure S4. Results of CCK-8 proliferation assays across various cell lines.

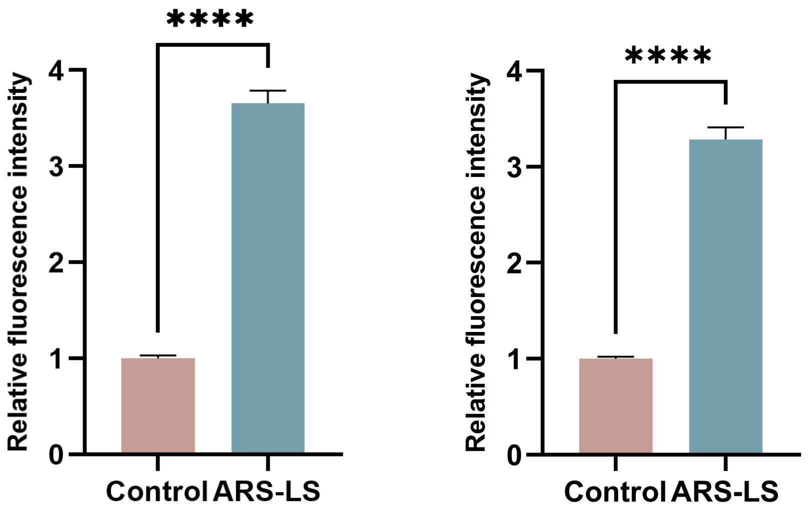

Figure S5. Quantitative analysis of relative fluorescence intensity in B16F10 cells and A375 cells after 20-minute incubation with FITC-labeled ARS and FITC-labeled ARS-LS.

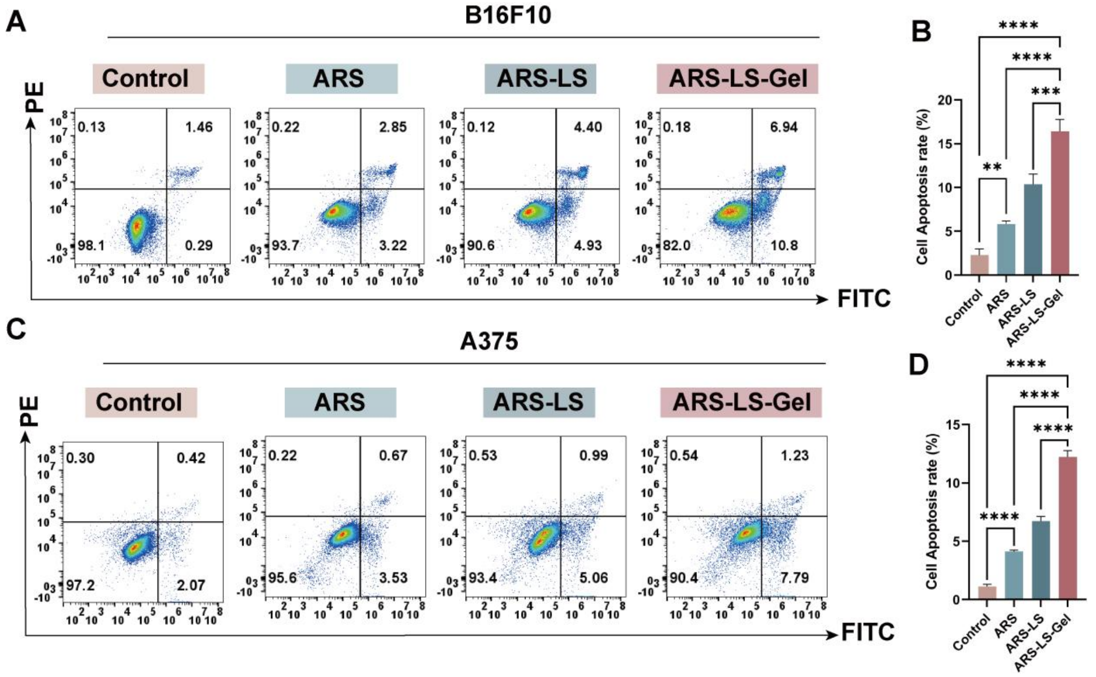

Figure S6. Comparative analysis of apoptosis rates by flow cytometry in B16F10 cells versus A375 cells following 24 h exposure to various drug formulations.

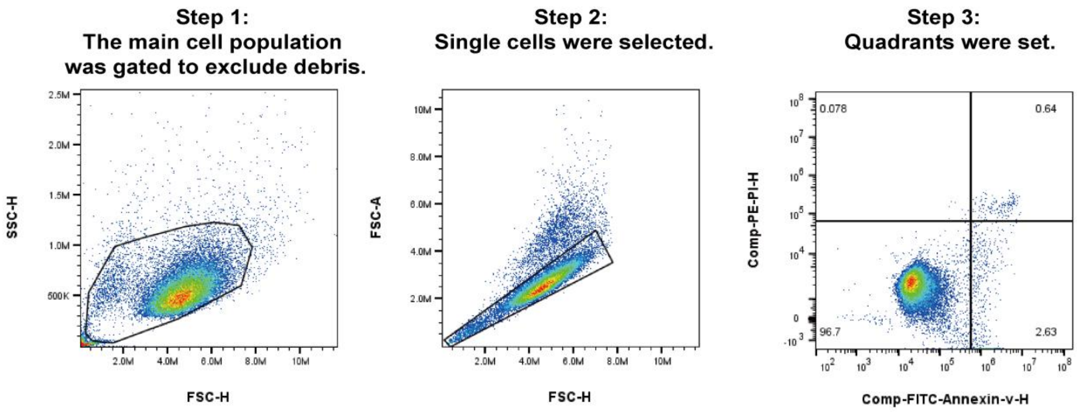

Figure S7. The gating strategy.

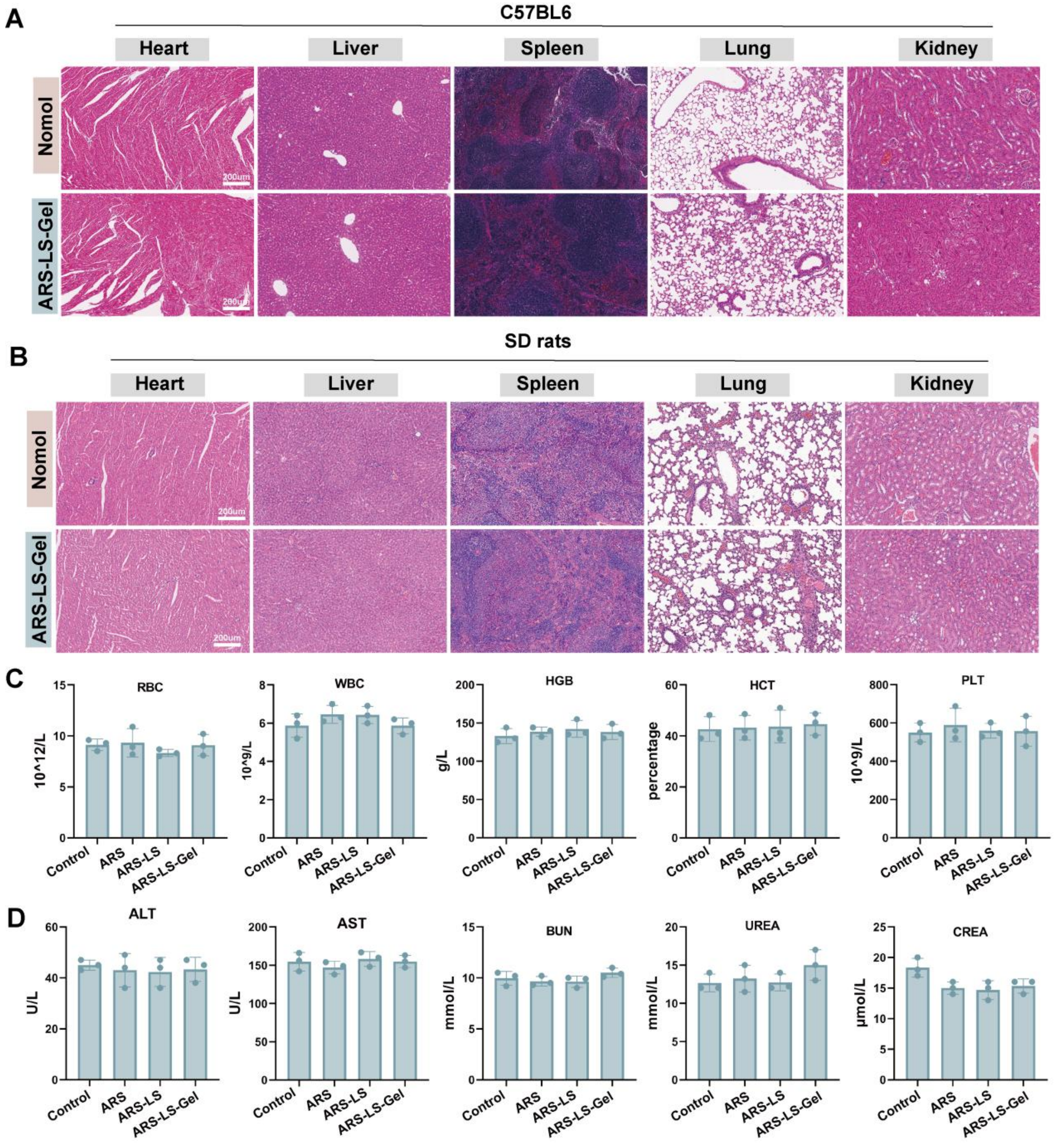

Figure S8. Histopathological and Hematological Safety Evaluation. (A-B) H&E staining of various organs (scale bar: 200 μm). (C) Complete blood panel analysis of

red blood cell (RBC), white blood cell (WBC), hemoglobin (HGB), hematocrit (HCT) and platelet (PLT), and serum biochemical (D) analysis of alanine aminotransferase (ALT), aspartate aminotransferase (AST), blood urea nitrogen (BUN), blood urea nitrogen (UREA), creatinine (CREA), data presented as mean ± SD (n = 3).

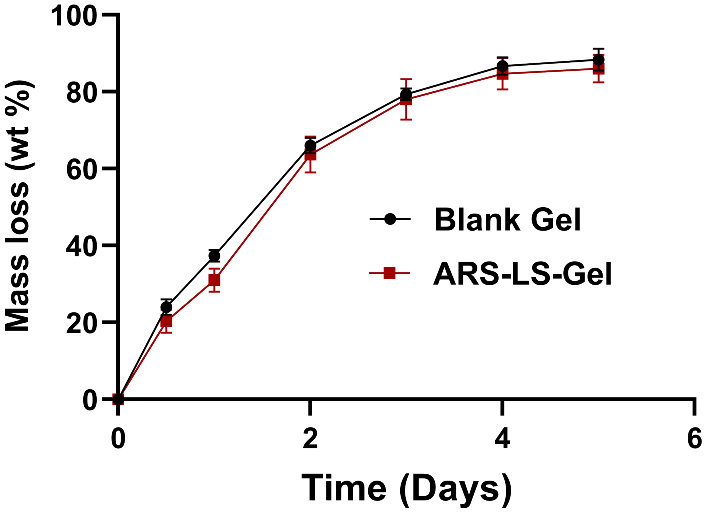

Figure S9. In vitro degradation analysis of blank hydrogel and ARS-LS-Gel.

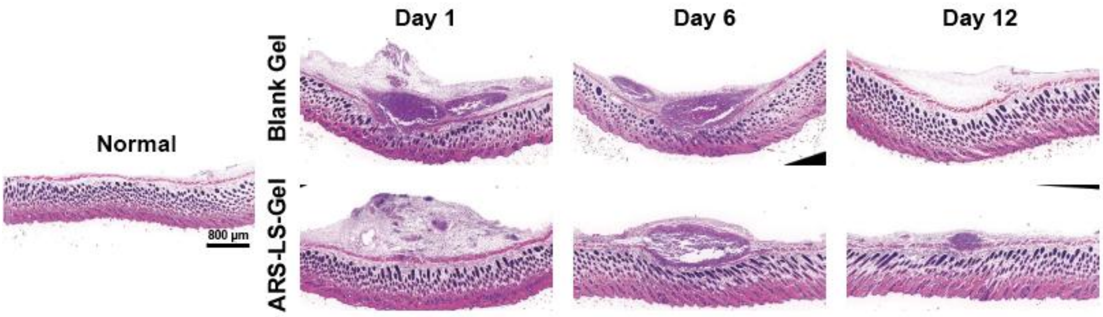

Figure S10. In vivo degradation test of blank hydrogel and ARS-LS-Gel.

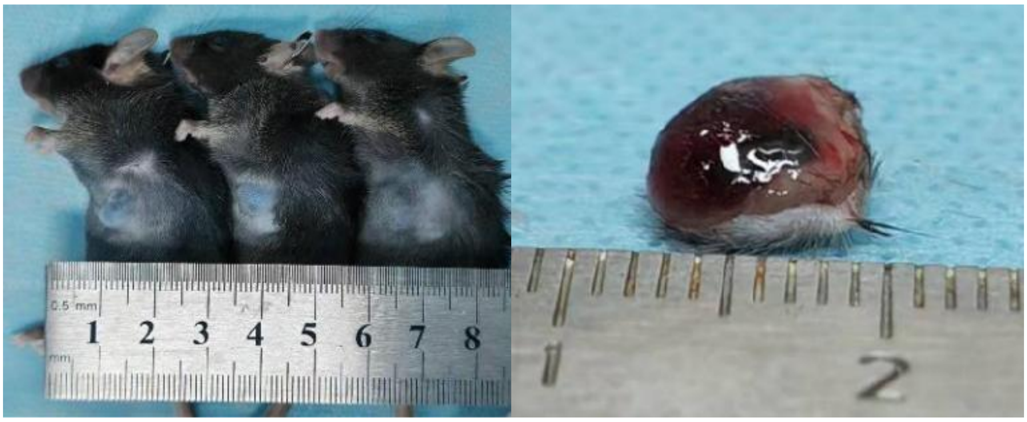

Figure S11. The tumor volume reached ~ 50 mm^3

Table S1. Primer sequences used in this study

Gene name	Primer Sequences
Bbc3-F	CAGCAGCACTTAGAGTCGCC
Bbc3-R	TGAGGGTCGGTGTCGATGCT
Cdkn1a-F	GGGACAAGAGGCCCAGTACT
Cdkn1a-R	CAATCTGCGCTTGGAGTGA
VDAC3-F	TACAGGGAAAGCATCAGGCAAC
VDAC3-R	CCCTTCAGCCAACTTATTCTCC
IL-1β-F	CAAGCAACGACAAAATCCC
IL-1β-R	CAAACCGCTTTTCCATCTTC
TNF-α-F	CCACGCTCTTCTGTCTACTG
TNF-α-R	GCTACGGGCTTGTCACTC
